# Easy on the Osteopath

**Published:** 1902-08

**Authors:** 


### EASY ON THE OSTEOPATH.

In commenting upon the new Massachusetts law which requires osteopaths to undergo examinations similar to those given to regular graduates in medicine, with the exception that they are to be *exempt from examinations in pathology and practice, American Medicine* says: "There could be no objection to licensing such candidates if they passed satisfactorily the same examinations as all others, but to except pathology and the practice of medicine is like excepting the Moor and Desdemona from the play of Othello. What is 'medicine' with pathology and treatment omitted? Where is the hole in the sand, asked the child, when you take the sand away? On such grounds and by such logic may not every street-corner peddler of wizard oil and magnetic ointment be permitted to practice 'medicine'? The belief that there can be more than one science of pathology should alone disbar the holder from practice."

The wonder is not that the law allows osteopaths to avoid having to qualify in the very essentials of medicine, but that it places any restrictions whatever upon them. Massachusetts long ago earned the reputation for being a tender mother to fakirs and fanatics of every description. It ill becomes her at this late day to put any stumbling block in the way of these osteopaths, or any other real simon pure humbugs.

Now that the bars have been even partially let down to admit the disciples of a fad almost as insane as her own, Mother Eddy had better look to her laurels. *Unchristian unscience* has lost the charm of novelty, while the new cult has the advantage of being both novel and absurd; qualities sure to win a large following in the old Bay State. R.

